# A small molecule Nec-1 directly induces amyloid clearance in the brains of aged APP/PS1 mice

**DOI:** 10.1038/s41598-019-40205-5

**Published:** 2019-03-12

**Authors:** Seung-Hoon Yang, Jisu Shin, Naewoo Neo Shin, Ji-Hyun Hwang, Sung-Chul Hong, Keunwan Park, Jae Wook Lee, Sejin Lee, Seungyeop Baek, Kyeonghwan Kim, Illhwan Cho, YoungSoo Kim

**Affiliations:** 1 Department of Medical Biotechnology, College of Life Science and Biotechnology, Dongguk University, Seoul, 04620 Republic of Korea; 2 Department of Pharmacy, Yonsei University, Incheon, 21983 Republic of Korea; 3 Yonsei Institute of Pharmaceutical Science, Yonsei University, Incheon, 21983 Republic of Korea; 4 Integrated Science and Engineering Division, Yonsei University, Incheon, 21983 Republic of Korea; 5 Natural Product Informatics Research Center, Gangneung, 25451 Republic of Korea; 6 Natural Constituent Research Center, Korea Institute of Science and Technology, Gangneung, 25451 Republic of Korea; 7 Department of Biotechnology, Yonsei University, Incheon, 21983 Republic of Korea

## Abstract

Alzheimer’s disease (AD) is a progressive neurodegenerative disorder characterized by the formation of toxic amyloid-β (Aβ) oligomers and plaques. Considering that Aβ misfolding and aggregation precedes the progressive development of cognitive impairment in AD, investigating a therapeutic means by clearance of pre-existing Aβ aggregates shows promise as a viable disease-modifying treatment. Here, we report that a small molecule, necrostatin-1 (Nec-1), reduces Aβ aggregates back to non-toxic monomers *in vitro* and *in vivo*. Intravenous administration of Nec-1 reduced the levels of Aβ plaques in the brains of aged APP/PS1 double transgenic mice. In addition, Nec-1 exhibited therapeutic effects against Aβ aggregates by inhibiting Aβ-induced brain cell death in neuronal and microglial cell lines. Nec-1 also showed anti-apoptotic and anti-necroptotic effects in the cortex of aged APP/PS1 mice by reducing levels of phosphorylated-RIPK3 and Bax and increasing the levels of Bcl-2. According to our data *in vitro* and *in silico*, the methyl group of the amine in the 2-thioxo-4-imidazolidinone is the key moiety of Nec-1 that directs its activity against aggregated Aβ. Given that the accumulation of Aβ aggregates is an important hallmark of AD, our studies provide strong evidence that Nec-1 may serve a key role in the development of AD treatment.

## Introduction

It is believed that deposition of amyloid-β (Aβ) aggregates is highly correlated to the pathogenesis of Alzheimer’s disease (AD)^1–4^. Aβ oligomers and plaques induce synaptic dysfunction and neuronal damage in the regions responsible for regulating learning and memory^2,5^. AD drugs used to be required, by US-FDA, to show improvements in patients’ cognitive functions. However, the criteria were revised and now drug candidates can use alteration of protein biomarkers such as Aβ for new endpoint of clinical trials. Because of these factors, Aβ aggregation has become a major target of AD drug candidates^6–9^. According to clinical studies, Aβ begins to accumulate in the brain at least a decade before onset of cognitive impairment^10^. Because of this buildup, preventative approaches cannot stop pre-existing Aβ from further damaging the brains of AD patients. Therefore, the most effective method of preventing further degradation would be to develop disease-modifying drugs that clear these pre-existing Aβ aggregates and aid in recovering cognition^11–14^. However, binding sites of Aβ aggregates for chemical drug candidates to are still unclear due to the limited information on structures of Aβ aggregates. On the contrary, it is relatively easier to develop immunotherapy drug candidates because Aβ aggregates can be used as antigens. This inhibits the development of designing an effective small molecule for the treatment of AD. Despite this, discovering small molecules will be more advantageous than antibody drugs because of their bioavailability, stability, and low-cost production^15,16^.

Necrostatin-1 (Nec-1) is a small molecule that inhibits necroptosis by regulating the activities of a protein complex formation containing receptor-interacting protein kinase 1 (RIPK1) and receptor-interacting protein kinase 3 (RIPK3)^17–20^. This RIPK1/RIPK3 complex was reported to exhibit structural characteristics of Aβ aggregates^21^. The protein assembly of RIPK1/RIPK3 forms heterodimeric fibrillar structure and shows classical characteristics of insoluble Aβ aggregates in structural investigations. Following studies showed evidences that necroptosis are associated with neuronal cell death in multiple neurodegenerative diseases and Nec-1 inhibit such degeneration in cellular and animal models of several brain disorders^22–25^. Our previous study on Nec-1 showed inhibitory and preventive effects on AD-like pathology and behavior. Nec-1 significantly blocked Aβ-induced neuronal cell death. When administered to adult APP/PS1 mice in a preventive manner before formation of protein aggregates and cognitive impairment, Nec-1 inhibited the development of Aβ and tau abnormalities^26^.

Based on our computational and biophysical evidences that Nec-1 binds to Aβ aggregates, we suspected that Nec-1 could also remove pre-existing Aβ aggregates in the brains of aged AD mice. In this study, we investigated the effects of Nec-1 on pre-existing Aβ aggregates *in vitro, in vivo*, and *in silico*. First, we added Nec-1 directly to a heterogeneous mixture of Aβ aggregates and monitored the levels of Aβ monomers, oligomers, and fibrils. Then, we investigated atomic details to reveal the key interactions between Nec-1 and Aβ responsible for Aβ plaque disaggregation. Afterwards, we examined the progressive effects of Nec-1 on Aβ aggregates-induced cell death of neuronal and microglial cell lines. To further assess therapeutic ability of Nec-1 for mouse study, Nec-1 was administered to amyloid-rich aged APP/PS1 mice by intravenous injection and changes in the levels of Aβ plaques were examined.

## Results

### Nec-1 disaggregates Aβ fibrils and oligomers and prevents brain cell death

**Figure 1:**
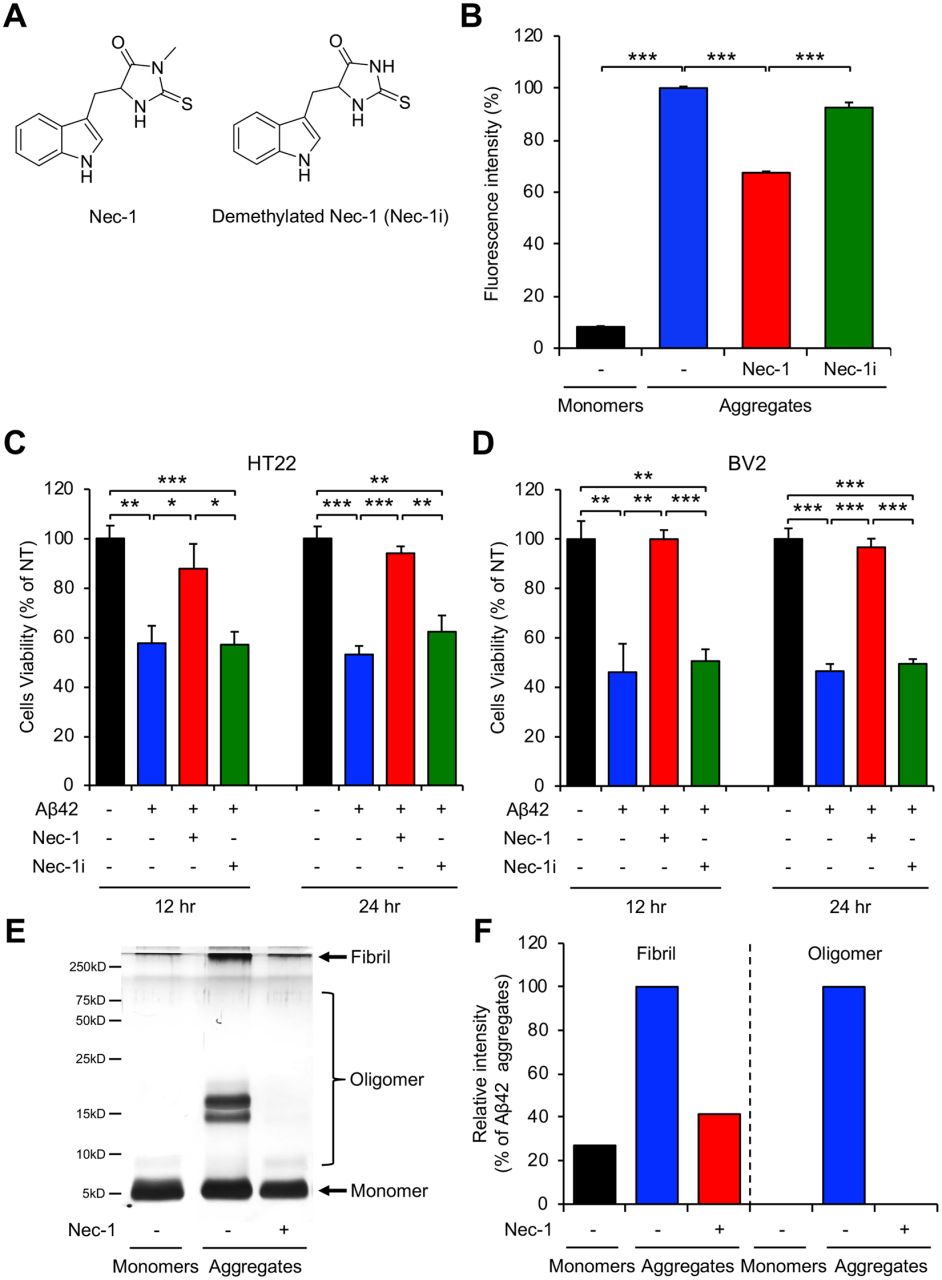
Nec-1 disaggregates synthetic Aβ aggregates. (**A**) Chemical structures of Nec-1 and demethylated Nec-1 (Nec-1i). (**B**) ThT assays for disaggregation of synthetic Aβ aggregates by two chemicals and only Nec-1 showed disaggregation effects. Fluorescence intensity was normalized to Aβ aggregates without compound treatments (100%). Cell viability assays using MTT in (**C**) HT22 neuronal cell line and (**D**) BV2 microglial cell line. Nec-1 or Nec-1i were treated to cells with pre-formed Aβ aggregates. Only Nec-1 inhibited Aβ-induced cell death. (**E**) Characterization and (**F**) quantification of Aβ42 peptides distribution by SDS-PAGE analysis with PICUP describing that Nec-1 reduced both oligomers and fibrils of to Aβ. All data presented are representative results of at least three independent experiments. Data is presented as mean ± SEM. *P ≤ 0.05, **P ≤ 0.01 and ***P ≤ 0.001 (One-way ANOVA followed by Bonferroni’s post-hoc comparison tests).

As AD gradually progresses, Aβ monomers aggregate into oligomers and fibrils^27^. Previously, we reported the inhibitory effect of Nec-1 on Aβ oligomerization and fibrillization^26^. In this study, we examined whether Nec-1 has an effect on disaggregation of pre-aggregated Aβ. For control, we synthesized demethylated Nec-1 (Nec-1i), which is known to be an inactive form of Nec-1 against RIPK^18^, to observe the molecular mechanism of Aβ disaggregation (Fig. 1A). We performed thioflavin-T (ThT) fluorescence assay to detect and quantify β-sheet complex of protein aggregates^28^. For Aβ aggregation, we incubated synthesized monomeric Aβ42 peptides at 37 °C for 5 days. Then, we added Nec-1 or Nec-1i to these Aβ aggregates and incubated for 5 more days. We found that Aβ fibrils were dramatically reduced in the presence of Nec-1, suggesting that Nec-1 may disaggregate pre-formed Aβ aggregates (Fig. 1B). Aβ is commonly known to cause brain cell death during the process of its self-aggregation into harmful Aβ oligomers^29^. MTT assay is conducted to assess the cytotoxicity and cell proliferation by measuring MTT cleavage of viable cells^30^. We added pre-formed Aβ42 aggregates and Nec-1 or Nec-1i to neuronal (Fig. 1C) and microglial cell (Fig. 1D) lines and incubated the cells for 12 or 24 hours. In the condition where Aβ aggregates would induce cell death, Nec-1 exhibits neuroprotective effects against Aβ aggregates. Collectively, we predict that such anti-cell death results were contributed by Nec-1’s ability to dissociate Aβ aggregates. Notably, the demethylation of 2-thioxo-4-imidazolidinone moiety disables the Aβ-disaggregating effects of Nec-1. It is suggested that Aβ oligomers are highly correlated with brain cell death. Thus, we wanted to observe whether Nec-1 could also disaggregate Aβ oligomers. We performed silver staining of SDS-PAGE with photo-induced cross-linking of unmodified proteins (PICUP) to visualize and compare the quantitative bands of Aβ species when treated with Nec-1. Both Aβ oligomers and fibrils were significantly reduced by Nec-1 (Fig. 1E,F). These results indicated that Nec-1 could disaggregate pre-existing synthetic Aβ aggregates such as oligomers and fibrils.

### Docking model differentiating Nec-1 from Nec-1i suggests key interactions to Aβ in atomic details

It was suggested in our previous work that Nec-1 specifically binds to Aβ^26^, and as such it may inhibit Aβ aggregation by competitively binding to hydrophobic patch in multimeric Aβ structures and prevent successive β-strand pairing. Additional experimental results on Nec-1i in this study enables us to propose more plausible docking models based on the assumption that removal of C10 in Nec-1 will substantially decrease binding affinity and reduce biological activity. The chemical structures of Nec-1 and Nec-1i are nearly identical except for the methyl group in C10.

In order to search for docking models that support our hypothesis, we performed exhaustive global (low-resolution) docking conformation search followed by high-resolution all-atom refinement. In brief, binding conformations of Nec-1 and Aβ were calculated using the PatchDock software^31^ with simplified structural representation but with very high speed and coverage to explore three-dimensional space without any prior knowledge on binding site. Resultant docking conformations were refined by an all-atom energy function of Rosetta molecular modeling suite^32^. A customized xml-based RosettaScript protocol was devised to obtain the final all-atom refined binding conformations.

**Figure 2:**
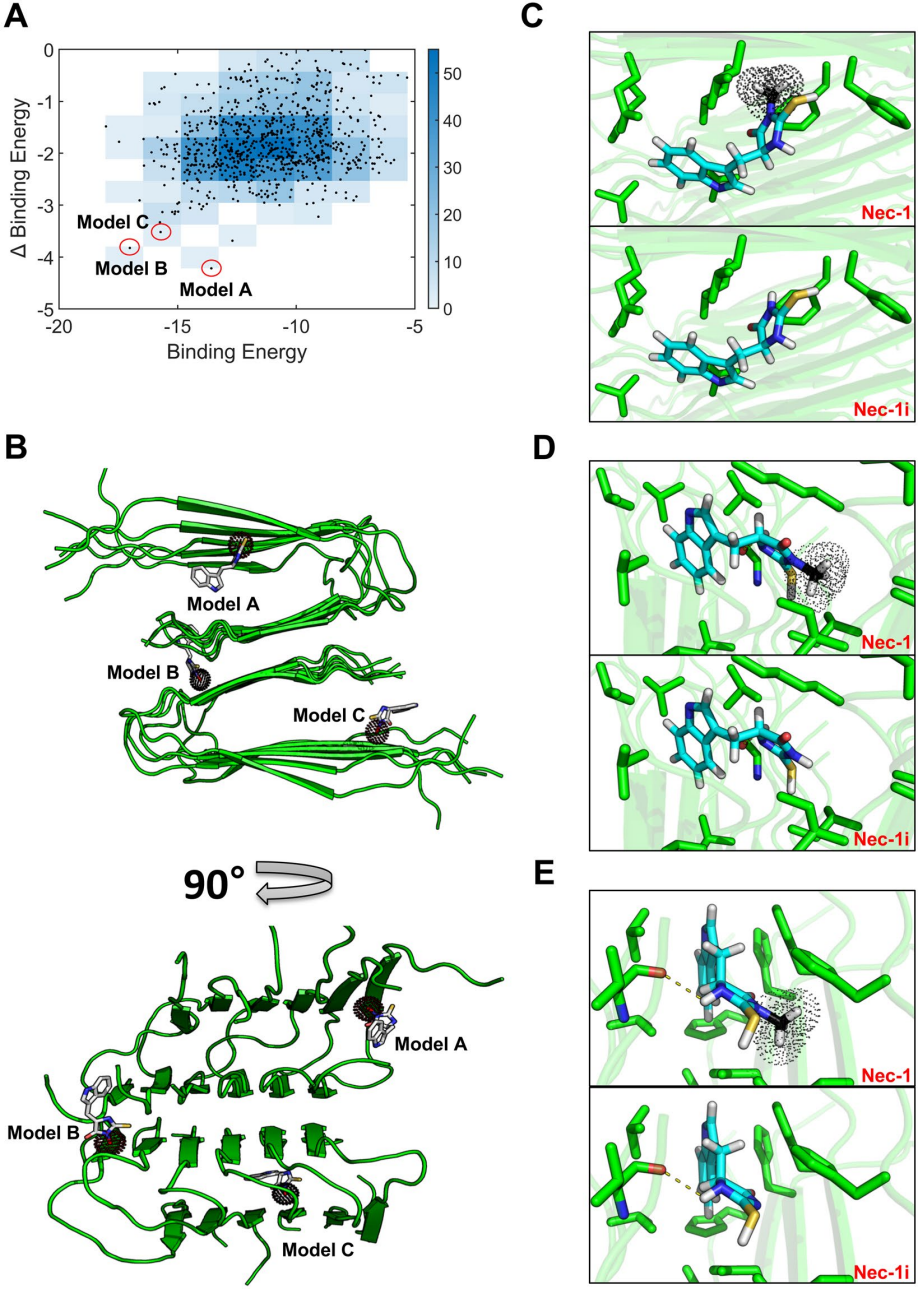
Docking model specific to Nec-1 suggests potential key interactions for binding to Aβ aggregates. (**A**) Docking models filtered by Rosetta energy (top 1%) were plotted in terms of calculated binding affinity (i.e. binding energy of Nec-1 to Aβ) and binding specificity (i.e. Δbinding energy between Nec-1 and Nec-1i). Three docking models of high binding affinity and specificity (model A, B and C marked by red circle) were selected as the most plausible Nec-1 binding models consistent with the experimental results. (**B**) Three docking conformations of Nec-1 were overlaid in Aβ structure, and the detailed Nec-1 or Nec-1i binding pose and its interacting residues within 5A distance for (**C**) model A, (**D**) model B, and (**E**) model C were shown by sticks where carbon is colored by cyan, nitrogen by blue, sulfate by yellow, oxygen by red, and hydrogen by white. C10 atom in Nec-1, absent in Nec-1i, is represented by black color with dots.

The docking experiments produced 839 models after filtering by Rosetta energy (−714.9 Rosetta Energy Unit - top 1% among pre-selected docking models). The selected 839 docking models were analyzed in terms of calculated binding affinity (binding energy of Nec-1, X-axis in Fig. 2A) and binding specificity to Nec-1i (Δbinding energy, binding energy difference between Nec-1 and Nec-1i, Y-axis in Fig. 2A). We finally selected three docking models (Fig. 2B) that have lower binding energy and, at the same time, have higher Δbinding energy than others (i.e. Pareto fronts in Fig. 2A), which are the most consistent with our experimental results.

Among the three docking models, Nec-1 in model A (the lowest Δbinding energy) is docked near the edge strand where it can effectively disturb Aβ aggregation. The C10 methyl group of Nec-1 (not found in Nec-1i) interacts with a few hydrophobic residues, whereas the bulky aromatic ring in Nec-1 (also found in Nec-1i) is attracted towards the opposite β-strands, showing that the lowest Δbinding energy is mainly originated from C10 methyl group (Fig. 2C). The results suggest that model A is one of the most plausible docking models that support the hypothesis on high binding specificity of Nec-1 over Nec-1i. Model B and C have slightly less favorable C10 interactions than model A but have lower overall binding energy due to more hydrophobic contacts (Fig. 2D,E). Nevertheless, they also show that Nec-1 binding can efficiently prevent formation of Aβ aggregation by interfering successive β-strand pairing because neither of them binds to an exposed β-strand surface. Despite the lower binding energy in model B and C, they have a large number of hydrophobic contacts apart from C10 as seen in the smaller Δbinding energy. Taken together, all the docking models presented provide atomic details that potentially reveal the key interactions between Nec-1 and Aβ.

### Nec-1 blocks Aβ aggregates-induced brain cell death

**Figure 3:**
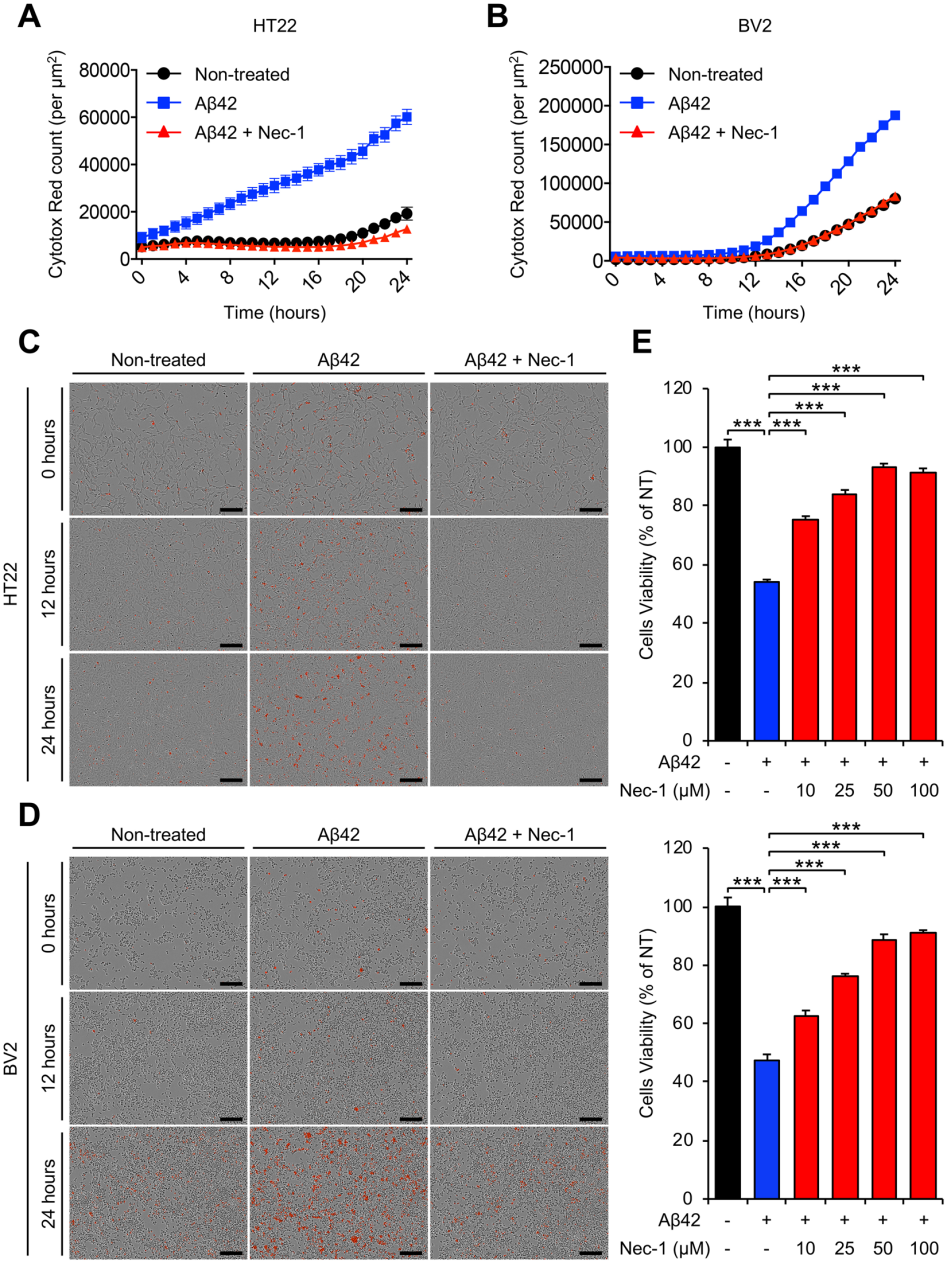
Nec-1 blocks brain cell death induced by Aβ aggregates. (**A**,**B**) Time course analysis of brain cell death measured by staining with IncuCyte Cytotox Red reagent in HT22 (**A**) and BV2 (**B**) cells. Nec-1 prevents Aβ-induced cell deaths (**C**,**D**) Representative images of the stained cells at indicated time point (C, HT22 cells; D, BV2 cells). Scale bar = 200 μm. (**E**) Dose-dependent cytotoxicity of Nec-1 on Aβ-induced cell death in HT22 (upper) and BV2 (lower) cells. Compared to Aβ only sample, Nec-1 treatment reduced the number of dead cells. Each cell line was seeded at equal amounts (5 × 10^3^ cells/well) into a 96-well plate. All experiments were performed twice. Data is presented as mean ± SEM. ***P ≤ 0.001 (One-way ANOVA followed by Bonferroni’s post-hoc comparison tests).

Our aforementioned experimental results indicate that Nec-1 has potential to protect cells against Aβ-induced neurotoxicity. Although Nec-1 can dissociate neurotoxic Aβ oligomers and fibrils into inert monomers, the Aβ species could re-aggregate due to their metastable nature^33^. To examine whether Nec-1 can maintain their neuroprotective effects, Aβ-induced neurotoxicity was observed in a time-dependent manner. We performed cell cytotoxicity assays using HT22 (hippocampal neuronal cell line) and BV2 (microglial cell line) cells. Each cell line was seeded at equal amounts (5 × 10^3^ cells/well) into a 96-well plate before addition of peptides and chemicals, Aβ42 and Nec-1. We prepared pre-existing Aβ42 aggregates by incubating synthetic Aβ42 monomer (1 mM) at 37 °C for 24 hours, and were then diluted with cell cultured medium. The cultured cells were treated with pre-existing Aβ42 aggregates (10 μM) in the presence or absence of Nec-1 (50 μM) for 24 hours. Cells cultured in absence of Aβ42 aggregates and Nec-1 (non-treated) were used as control. For kinetic monitoring and real-time imaging of cell cytotoxicity, we conducted the IncuCyte^®^ Zoom analysis. In this analysis, dead cells were distinguished from live cells by staining with IncuCyte^TM^ Cytotox Red reagent. In both HT22 and BV2 cells, the Cytotox Red counts indicated that dead cells were increased with treatment of Aβ42 aggregates in time-dependent manner. However, the levels of cell death by Aβ42 aggregates were dramatically decreased for cells treated with Nec-1 (Fig. 3A,B). In real-time imaging, we found that the dead cells induced by Aβ42 aggregates were greatly reduced by treatment of Nec-1 in both cells, although endogenous cell deaths were also shown even in non-treated cells (Fig. 3C,D). In addition, Nec-1 significantly inhibited the level of cell death induced by Aβ42 aggregates in a dose-dependent manner on HT22 and BV2 cells (Fig. 3E). Collectively, these findings suggested that Nec-1 blocks pre-existing Aβ aggregates-induced brain cell death and maintains neuroprotection.

### Nec-1 reduces Aβ plaques in the brains of aged APP/PS1 mice

**Figure 4:**
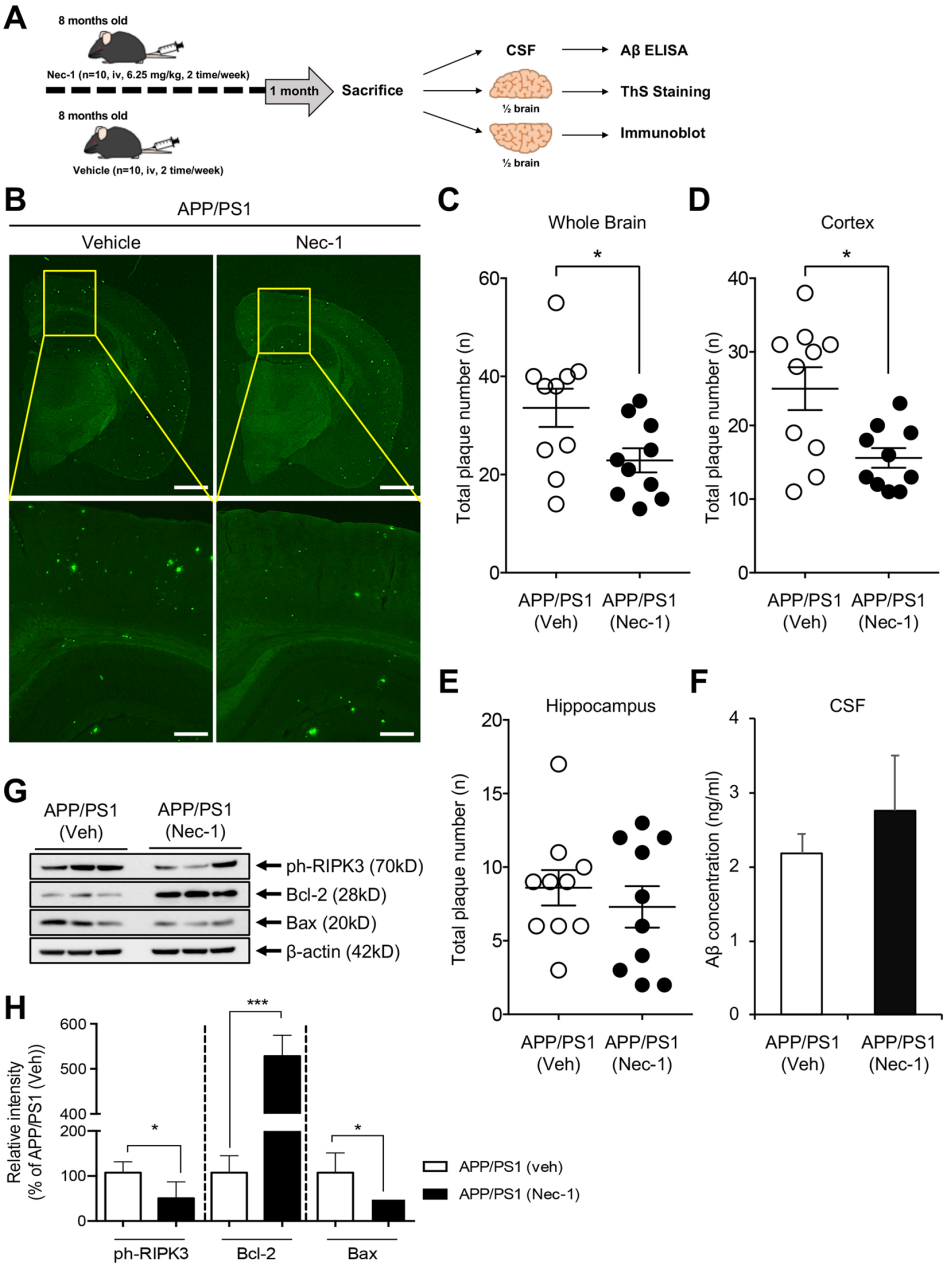
Nec-1 reduces Aβ plaques in aged APP/PS1 mouse brains. (**A**) Schedule of Nec-1 administration. Nec-1 (6.25 mg/kg, *n* = 10) or vehicle (2.5% DMSO in PBS, *n* = 10) was injected into 8-month-old male APP/PS1 mice via tail vein for 4 weeks (2 times per week). Brains and CSF samples were collected after sacrifice. The illustration was drawn using Adobe Photoshop software program. (**B**) ThS-stained Aβ plaques in whole brains of APP/PS1 mice treated with vehicle (n = 10) or Nec-1 (n = 10). Data presented in this article are 4 representative images. Scale bars = 1 mm (upper), 200 μm (lower). Total numbers of ThS-positive Aβ plaques in whole brain (**C**), cortex (**D**), and hippocampus (**E**) of APP/PS1 mice after Nec-1 administration. Numbers of plaques were reduced by Nec-1 administration. (**F**) Aβ42 levels in cerebrospinal fluid from five mice which used for ThS staining in aforementioned mice with or without Nec-1 treatment. (**G**) Western blot analysis and (**H**) quantification of phosphorylated RIPK3, Bcl-2, and Bax to observe changes of necroptosis and apoptosis. Three mice from each group were analyzed for this experiment. Phosphorylated RIPK3 and Bax were reduced, while Bcl-2 was increased by Nec-1 administration. Data is presented as mean ± SEM. *P ≤ 0.05 and ***P ≤ 0.001 (One-way ANOVA followed by Bonferroni post-hoc comparison tests). Full-length original blots are shown in Supplementary Information.

The disaggregating effects of Nec-1 on Aβ aggregates suggest a potential application of AD treatment during the later stages of AD, where Aβ plaques have already formed. We examined the effects of Nec-1 on pre-existing Aβ plaques using aged APPswe/PS1-dE9 double-transgenic mouse models (APP/PS1). This mouse model expresses mutant human amyloid precursor protein (APP) and presenilin protein 1 (PS1), allowing development of Aβ plaque formation as early as 4 months of age, with abundant plaques in the brain by 9 months of age^34,35^. Nec-1 (6.25 mg/kg/mouse) or vehicle only was intravenously administered (2 times/week) to 8-months-old APP/PS1 mice (*n* = 10/group, male) for 4 weeks until the age of 9 months (Fig. 4A). After the 4-week injection, brains and CSF of each mouse in all subject group were collected after sacrifice for the further study. To detect insoluble β-sheet-rich Aβ plaques, the cryosections of brain tissues were stained with thioflavin S (ThS)^36^. We found that plaques were reduced in the brains of Nec-1-injected mice compared to those of vehicle-injected APP/PS1 mice (Fig. 4B). Total plaque numbers in whole brains and cortical regions of Nec-1-administered APP/PS1 mice were significantly decreased than those administered with vehicles; however, there were no significant differences in the hippocampal regions (Fig. 4C–E). These results confirmed that Nec-1 administration could reduce Aβ plaques that were already formed in the brain of aged APP/PS1 mice. Different from immunotherapy, where Aβ is cleared in the brain, Nec-1 only dissociates oligomers and plaques. If these dissociated species stay in the brain, there is a possibility for them to re-aggregate. Thus, these species must be secreted from the brain to bypass the concern that Aβ monomers can build up into oligomers and plaques in the brain again. The cerebrospinal fluid samples of mice from the aforementioned experiments were collected and the concentration of Aβ levels were analyzed by sandwich ELISA (KHB3544). The results show that Nec-1-treated APP/PS1 mice do not have different levels of Aβ compared to the vehicle group (Fig. 4F). Apoptosis in the cortical regions of the brain is known to be correlated with symptoms of AD^19,37^. Thus we examined if the disaggregation of plaques influenced apoptosis in the brain of APP/PS1 mice by Nec-1 injection. Cortical region of the APP/PS1 brain were homogenized and lysed. Then they were assessed by immunoblotting to check the expression levels of necroptotic and apoptotic molecules (Fig. 4G,H). Nec-1 decreased necroptosis and apoptosis in the cortex of aged APP/PS1 mice by reducing levels of phosphorylated-RIPK3 and Bax and increasing the levels of Bcl-2. Taken together, intravenous administration of Nec-1 to aged APP/PS1 mice disaggregated Aβ plaques and reduced apoptosis in the cortex of brain.

## Discussion

Here we report that Nec-1 disaggregates Aβ oligomers and plaques back into non-toxic monomers *in vitro* and *in vivo*. Such Aβ-disaggregating ability of Nec-1 also protects neuronal and microglial cell death induced by Aβ aggregates. The targets of Alzheimer drug candidates have been shifted from preventively regulating the production or aggregation of Aβ to amyloid clearance from the brain, and significant results from clinical trials of Aducanumab support the stance that removal of Aβ aggregates confers clinical benefits. Previously, it was considered that such mode of action is only limited to immunotherapy. Here we provide strong evidence that the small molecule Nec-1 shares mode of action with Aducanumab in targeting and clearing Aβ aggregates^11^.

Although the evidences are limited to preclinical levels, Nec-1 has additional therapeutic mechanisms such as reducing hyperphosphorylation and aggregation of tau^26^. Altogether, our findings suggest that Nec-1 is a promising small molecule drug candidate for AD. Additional studies are warranted to determine whether the use of Nec-1 will translate into medicine that may benefit AD preventatively and therapeutically. Notably, we observed that the demethylated form of Nec-1 does not affect Aβ, which suggests that the 3-methyl-2-thioxo-4-imidazolidinone structure may serve as a targeting or disaggregating moiety. Our current finding further supports the hypothesis that RIPK complex formation shares amyloidogenic similarities with Aβ aggregates^21,26^. Nec-1 was reported to have about 1–2 hour of half-life with bioavailability of 54.8% in rats^38^. Therefore, stability of Nec-1 needs significant improvements for Nec-1 or its derivatives to become an orally available drug. However, numbers of study already provided experimental evidences that Nec-1 can penetrate blood-brain barrier affect biomarkers in the brain of animal models.

Transgenic mouse models typically do not reflect clinical cases in terms of atrophy in the brain; therefore, mice are not perfect models to study cognitive alterations by Aβ aggregates-targeting drug candidates. Larger animals, such as TgF344-AD transgenic rats, need to be utilized to further characterize the Aβ-clearing action of Nec-1 and its resulting effects on cognition^39^. Although Nec-1 is known to disrupt complex formation of diverse proteins such as receptor-interacting protein kinase and amyloids, the detail mechanism remains unclear. Further research on the biophysical properties and derivatives of Nec-1 will provide novel insights on drug development for AD.

## Materials and Methods

### Reagents

Aβ42 peptides were synthesized by following the DMSO-incorporated Fmoc solid phase peptide synthesis (SPPS) protocol^40^. Necrostatin-1 (Nec-1) and thioflavin S (ThS) were bought from Sigma-Aldrich. IncuCyte^TM^ Cytotox Red reagents for counting dead cells were purchased from ESSEN Bioscience. The antibodies used for immunoblotting were anti-ph-RIPK3 (Catalog ab209384, Abcam), anti-Bax (Catalog #2772, Cell Signaling Technology), anti-Bcl-2 (Catalog #2876, Cell Signaling Technology), anti-β-actin (Catalog MAB1501, Millipore Corporation).

### Synthesis of demethylated Nec-1 (Nec-1i)

The synthesis of 5-(1H-indol-3-ylmethyl)-2-thioxo-4-imidazolidinone has previously been described^41^.

### Aβ42 disaggregation assay

Aβ42 solutions (25 μM) were made by dissolving in-house synthetic Aβ42 peptides (25 mM) in DMSO and then diluted with deionized water. After incubating Aβ42 solutions for 5 days at 37 °C to induce aggregation, Nec-1 (500 μM) was added. The mixed solutions were re-incubated for an additional 5 days. Thioflavin T (ThT) assay was used to observe Aβ aggregation. ThT (5 μM in 50 mM glycine buffer, pH 8.9) was added in 96-well black plate and incubated for 3 hours. EnSpire® Multimode Plate Reader (Perkin-Elmer) was used to detect the fluorescence of Aβ-bound ThT at 450 nm (excitation) and 485 nm (emission).

### SDS-PAGE with photo-induced cross-linking of the unmodified proteins (PICUP)

SDS–PAGE and PICUP chemistry were conducted to evaluate Aβ species by size distribution^42^. Aβ peptides were dissolved in DMSO as 10 mM stocks. Stocks were then diluted 40-fold by PBS and incubated for 5 day in 37 °C to induce aggregation. To induce cross-linking, pre-aggregated Aβ solution were mixed with 1 mM Ru(Bpy)(Cl2) and 20 mM ammonium persulfate dissolved in 0.1 M sodium phosphate buffer (pH 7.4). After twice irradiation (each session for 1 second), cross-linked Aβ samples were analyzed on 15% tris-tricine gels, where they are separated into bands and visualized with silver staining.

### Cell culture

HT22 and BV2 cells were bought from the Korean Cell Line Bank (Seoul National University, Republic of Korea) and cultured in DMEM media (Supplemented with 10% (vol/vol) fetal bovine serum (Gibco), 100 units/mL penicillin, and 50 μg/mL streptomycin). Cells were seeded at a proper density of cells in fresh culture medium and maintained at 37 °C in a humidified 5% CO_2_ incubator.

### MTT cell viability assay

To examine the effect of Nec-1 or Nec-1i on Aβ-induced brain cell death, HT22 and BV2 cells were assessed using MTT cytotoxicity assay as previously reported^13^. Cultured cells were plated into a 96-well plate (5 × 10^3^ cells/well). Aβ42 solutions dissolved in DMSO (10 mM) were diluted 10 times using cell starvation medium (0.5% fetal bovine serum in DMEM) and then incubated for 24 hours at 37 °C for aggregation. After treatment of pre-aggregated Aβ42 (10 μM), Nec-1 (10, 25, 50, 100 μM), and demethylated Nec-1 (Nec-1i, 50 μM), MTT reagent (15 μM) was added to each well and was re-incubated for 4 more hours. Solubilization solution was then added and the plate was kept in room temperature overnight. The insoluble formazan was measured using Synergy^TM^ HT Multi-Detection microplate reader (Bio-Tek).

### Generation of docking models differentiating Nec-1 from Nec-1i

Initial two-dimensional structure of Nec-1 was obtained from Pubchem (CID 2828334) and its three-dimensional conformers were generated using Discovery Studio software^43^. Conformer generation was performed by ‘BEST’ algorithm followed by CHARMM force field minimization, which resulted in six distinct conformers all used in the subsequent docking procedure as a separate input molecule. The structure of Aβ (PDB ID 2LMO) was prepared as described in our previous work^26^.

The overall docking procedure largely consists of low-resolution global docking conformation search and all-atom local refinement. Global docking conformation search using the PatchDock software calculates high shape-complementarity docking poses by surface patch matching^31^ to estimate a rough Nec-1 binding conformation, which can be further refined by Rosetta all-atom energy. Output docking structures from PatchDock were then used as a starting conformation to apply customized Rosetta ligand docking and minimization protocol^44^. Specifically, 3,000 docking conformations were considered for all six conformers from the initial global conformation search (i.e. top 500 conformations per each Nec-1 conformer), and were then refined by using RosettaScript. This extensive all-atom local refinement by Rosetta is composed of two rounds of low-repulsive docking/minimization followed by three rounds of normal docking/minimization. As a result after 100 refinement trials of each docked conformation, 300,000 models were finally generated and evaluated by Rosetta energy, Nec-1 binding energy, and Δbinding energy between Nec-1 and Nec-1i. To elaborate, models that have positive Δbinding energy (i.e. lower Nec-1i binding energy) and positive ligand energy difference (i.e. lower Nec-1i total energy) were filtered out first, and then only the top 1% docking models in terms of Rosetta energy (total score term -714.9, Rosetta Energy Unit) were considered to get rid of poor energy conformations. The remaining models were then examined by Nec-1 binding energy and Nec-1/Nec-1i Δbinding energy. A binding energy of Nec-1i corresponds to that of Nec-1 and calculated by identical Rosetta energy function directly from Nec-1i structure removing C10 atom and restoring hydrogens (by Openbabel^45^) from Nec-1. The most plausible docking models showing favorable Nec-1 and unfavorable Nec-1i binding were selected after manually inspecting Pareto frontiers from the two energy variables shown in Fig. 2.

### Time-dependent cytotoxicity assay

HT22 and BV2 cells were seeded at equal amounts (5 × 10^3^ cells/well) into a 96-well plate before treating with Aβ42 and Nec-1. Aβ42 solutions were pre-aggregated as aforementioned. Aggregated Aβ42 (10 μM) and Nec-1 (50 μM) were added to the HT22 and BV2 cells and re-incubated for an additional 24 hours. Cell viability for kinetics and real-time imaging were assessed by measuring IncuCyte^TM^ Cytotox Red fluorescence using IncuCyte^®^ Zoom (Essen Bioscience) according to the manufacturer’s instructions.

### Animals

APP/PS1 double transgenic mice (male, APP/PS1, strain name; B6.Cg-Tg (APPswe, PS1dE9) 85Dbo/J) were purchased from Jackson Laboratory (Bar Harbor, Maine, USA) and raised in a laboratory animal breeding room at Korea Institute of Science and Technology under controlled temperature with an alternating 12 hours light-dark cycle. Food and water were available ad libitum. The genotypes of APP/PS1 mice were confirmed by PCR analysis of tail DNA following the standard protocol recommended by the Jackson Laboratory. Twenty APP/PS1 mice were assessed at the age of 8 months in this study. Nec-1 (n = 10, male) or vehicle (n = 10, male) was intravenously administered into 8-months-old male APP/PS1 mice for 4 weeks until the age of 9 months (Fig. 4A). Mice were then anesthetized by intraperitoneal injection of 4% avertin (400 mg/kg) and then perfused with 0.9% NaCl. The isolated brains were used in half for ThS staining and immunoblot analysis. All animal experiments were carried out in accordance with the National Institutes of Health guide for the care and use of laboratory animals (NIH Publications No. 8023, revised 1978). The animal experiments were conducted with authorization from the Institutional Animal Care and Use Committee of Korea Institute of Science and Technology.

### ThS staining

Nec-1 (6.25 mg/kg) or vehicle were administered to 8-month old mice (10 mice for each group) via tail vein twice a week for four weeks as described in Fig. 4A. The excised brains of APP/PS1 mice were fixed in 4% paraformaldehyde (pH 7.4) for 16 hours. The fixed brain samples were then immersed in 30% sucrose for cryoprotection and cut into 35 μm thick slices using a Cryostat (Microm HM 525, Thermo Scientific, Waltham, MA, USA). To visualize Aβ plaques, the sliced brains were stained with thioflavin S (ThS) for 7 minutes. 500 μM of thioflavin S (ThS) was dissolved in 50% ethanol. After rinsing with 100%, 95%, and 70% ethanol successively, the sections were moved into PBS. Images were taken with a Leica DM2500 fluorescence microscope. The plaque numbers were calculated from single brain image of each mice by using ImageJ software program.

### Cerebrospinal fluid analysis

All cerebrospinal fluid samples were collected via the cisterna magna^46^. Mice were anesthetized with a 4% avertin (400 mg/kg, IP) and were placed on a horizontal surface. A capillary tube was inserted into the surgically opened cisterna magna of mice to collect the cerebrospinal fluid. After collecting the sample, they were then stored at −80 °C until use. Aβ42 levels in cerebrospinal fluids of Nec-1-administered 8-month old APP/PS1 mice were compared to vehicle-administered APP/PS1 mice using Aβ42 sandwich ELISA kit from Invitrogen (KHB3544). For the measurement of Aβ(1–42) in cerebrospinal fluids, all cerebrospinal fluid samples were diluted 50-fold with standard diluent buffer. Then ELISA analysis was conducted according to the manufacturer’s instructions.

### Immunoblot analysis of brain lysates

Cortical regions of three brain from each experimental group were dissected and homogenized in ice-cold RIPA buffer (20 mM Tris-HCl, pH 7.5, 50 mM NaCl, 0.5% NP-40, 4 mM EDTA, 0.1% SDS, 0.5% sodium deoxycholate, and protease inhibitor cocktail). Homogenized brain tissues were then incubated in ice for 15 minutes before centrifugation at 14,000 rpm at 4 °C for 30 minutes. The supernatants of brain lysates were used for immunoblot analysis after quantification of concentrations by BCA assay. The brain lysates (20 μg) were subjected to reduced SDS-PAGE with 12% gel and were transferred to nitrocellulose membrane. After blocking with 5% skim milk in PBST, membranes were treated with primary antibodies (1:100 dilution in PBS for all antibodies) for overnight at 4 °C and were then detected by horseradish peroxidase-conjugated anti-mouse or anti-rabbit IgG antibodies. The blots were developed with SuperSignal West Pico substrate (Thermo Scientific) according to the manufacturer’s instructions. Blot density was quantified with normalization to β-actin by using ImageJ software program.

### Statistical analysis

All graphs were obtained with GraphPad Prism 6.0 software, and all statistical analyses were conducted with one-way ANOVA followed by Bonferroni’s post-hoc comparisons (*P < 0.05, **P < 0.01, ***P < 0.001). The error bars represent the SEMs.

## Supplementary information

Supplementary information
