# Supplementary material for: A small molecule Nec-1 directly induces amyloid clearance in the brains of aged APP/PS1 mice
Source: Sci Rep. 2019 Mar 12;9:4183. doi: 10.1038/s41598-019-40205-5 (PMC6414664; doi:10.1038/s41598-019-40205-5)
Supplement: Supplementary file 1 — Supplementary information [file 41598_2019_40205_MOESM1_ESM.pdf]

Supplementary Information

A small molecule Nec-1 directly induces amyloid clearance in the brains of aged APP/PS1 mice.

Seung-Hoon Yang[1], Jisu Shin[2,3], Naewoo Neo Shin[4], Ji-Hyun Hwang[5], Sung-Chul Hong[5], Keunwan Park[5], Jae Wook Lee[6], Sejin Lee[2,3], Seungyeop Baek[2,3,7], Kyeonghwan Kim[2,3], Illhwan Cho[2,3] & YoungSoo Kim[2,3,4,*]

[1]Department of Medical Biotechnology, College of Life Science and Biotechnology, Dongguk University, Seoul 04620, Republic of Korea

[2]Department of Pharmacy, [3]Yonsei Institute of Pharmaceutical Science, and [4]Integrated Science and Engineering Division, [7]Department of Biotechnology, Yonsei University, Incheon 21983, Republic of Korea

[5]Natural Product Informatics Research Center and [6]Natural Constituent Research Center, Korea Institute of Science and Technology, Gangneung 25451, Republic of Korea

*Correspondence and requests for materials should be addressed to Y.K. (y.kim@yonsei.ac.kr)

Supplementary figure legends

Supplementary figure S1. Original gel blot of ph-RIPK3 (A), Bcl-2 (B), Bax (C), β-actin (D) presented in Figure 4G.

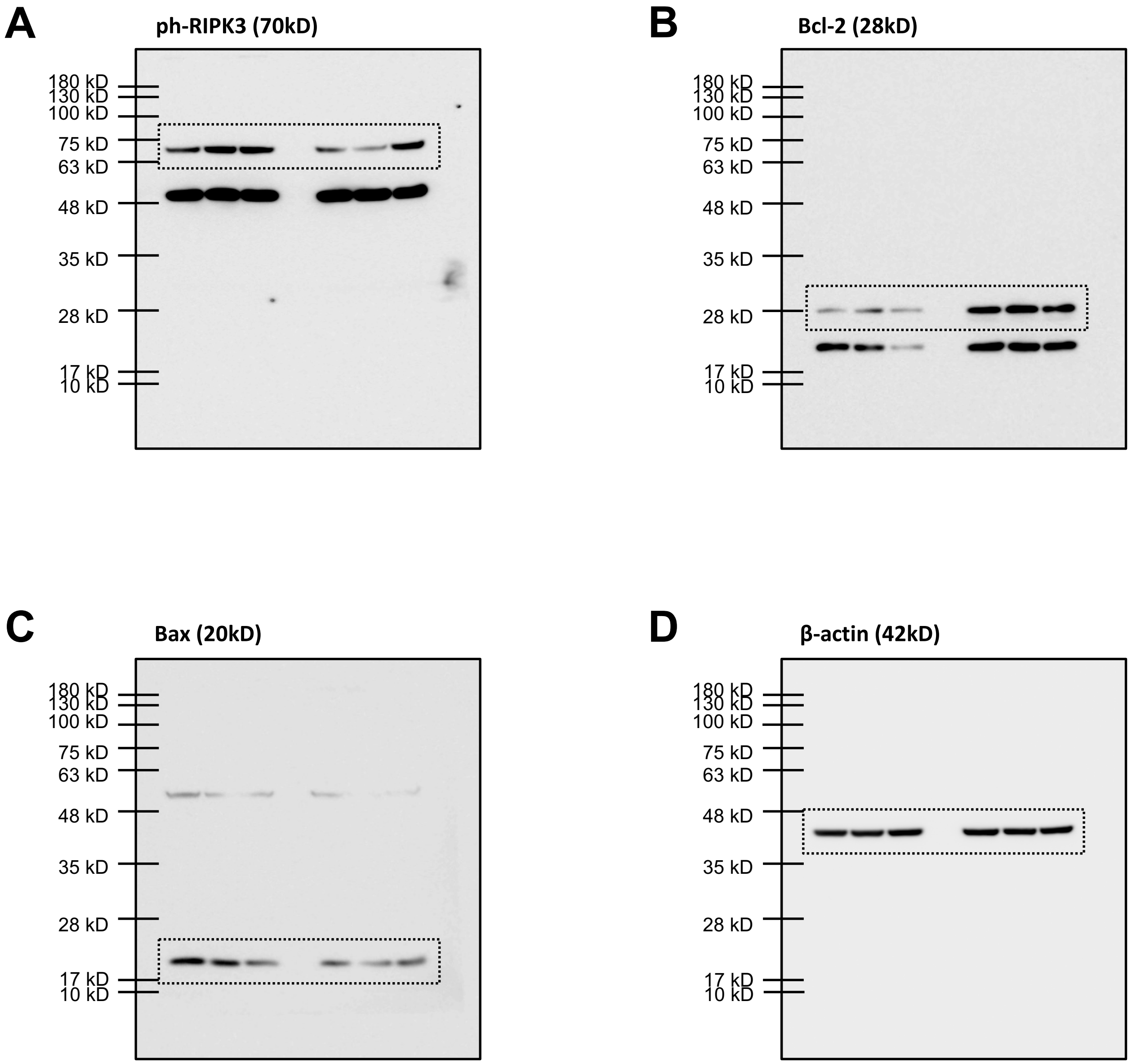
A
ph-RIPK3 (70kD)
180 kD
130 kD
100 kD
75 kD
63 kD
48 kD
35 kD
28 kD
17 kD
10 kD
B
Bcl-2 (28kD)
180 kD
130 kD
100 kD
75 kD
63 kD
48 kD
35 kD
28 kD
17 kD
10 kD
C
Bax (20kD)
180 kD
130 kD
100 kD
75 kD
63 kD
48 kD
35 kD
28 kD
17 kD
10 kD
D
β-actin (42kD)
180 kD
130 kD
100 kD
75 kD
63 kD
48 kD
35 kD
28 kD
17 kD
10 kD
